# Comprehensive sexuality education linked to sexual and reproductive health services reduces early and unintended pregnancies among in-school adolescent girls in Zambia

**DOI:** 10.1186/s12889-023-15023-0

**Published:** 2023-02-16

**Authors:** Michael T. Mbizvo, Kondwani Kasonda, Nelly-Claire Muntalima, Joseph G. Rosen, Sophie Inambwae, Edith S. Namukonda, Ronald Mungoni, Natasha Okpara, Chifundo Phiri, Nachela Chelwa, Chabu Kangale

**Affiliations:** Population Council, Lusaka, Zambia

**Keywords:** Comprehensive sexuality education, Adolescent girls and young women, Early and unintended pregnancies, Adolescent sexual and reproductive health, Sub-Saharan Africa

## Abstract

**Background:**

Advancing the health of adolescents, particularly their sexual and reproductive health, including HIV prevention and care, is a development imperative. A critical part for improving their wellbeing and economic development is the social status accorded to adolescent girls and young women (AGYW). However, AGYW in many countries including Zambia, encounter health challenges that stem from gender inequalities, lack of empowerment, inaccurate knowledge on sexuality, and poor access to sexual and reproductive health (SRH) services and information. Addressing the knowledge gaps through comprehensive sexuality education (CSE) and improving access to SRH services and appropriate information, should reduce school attrition from early and unintended pregnancies (EUP) and enhance realization of their full potential.

**Methods:**

The aim was to reduce EUP and improve SRH outcomes among AGYW in Zambia through provision of CSE linked to receptive SRH services. A 3-Arm randomized control study collected cross-sectional data at baseline, midline and Endline. Schools where CSE was being routinely provided were randomized into a non-intervention arm (arm1), an intervention arm in which information on available SRH services was provided in schools by health workers to complement CSE, (arm 2), and arm 3 in which pupils receiving CSE were also encouraged or supported to access pre-sensitized, receptive SRH services.

**Results:**

Following 3 years of intervention exposure (CSE-Health Facility linkages), findings showed a significant decline of in-school pregnancies amongst AGYW in both intervention arms, with arm two exhibiting a more significant decline, having recorded only 0.74% pregnancies at endline (*p* < 0.001), as well as arm 3, which recorded 1.34% pregnancies (*p* < 0.001). No significant decline was recorded in the CSE only control arm. Trends in decline of pregnancies started to show by midline, and persisted at endline (2020), and when difference in differences test was applied, the incident rate ratios (IRR) between the none and exposed arms were equally significant (*p* < 0.001).

**Conclusion:**

Linking provision of CSE with accessible SRH services that are receptive to needs of adolescents and young people reduces EUP, which provides the opportunity for higher retention in school for adolescent girls.

## Plain English summary

Among Zambia’s key health and development challenges, are high rates of EUP, and disproportionately higher HIV rates among AGYW. Pregnancy among girls in school poses a challenge. CSE programmes are part of available armamentarium to improve knowledge on the risks. Poor SRH places a heavy strain on health systems and undermines sustainable development efforts. In response to these challenges, we initiated implementation research to develop and test a model linking CSE programs in schools with access to SRH services and information. Research was conducted in 23 schools and their local catchment health care facilities in two North Western province districts of Zambia. Following baseline data collection, schools were selected to ensure comparability of indicators such as reported pregnancy rates, CSE, and subsequently randomized into three study arms, with the first arm continuing standard instruction of CSE (control). The second arm was designed to bring information on available SRH services to schools, including raising awareness through health fares and clubs. In the third arm, health providers were trained to be more receptive to ASRH health needs, in addition to encouraging teachers to introduce students to health services. Pregnancies declined in all intervention arms at endline, with marked reductions in intervention Arm 2 which recorded 0.74% of in school pregnancies (*p* < O.001), followed by arm 3, with 1.34% (*p* < 0.001). The decline in intervention arms was more than 50% in intervention arms, as compared with control arm, and significantly lower, as a proportion of AGYW in arm 2, followed by arm 3.

## Background

School attrition attributed to early and unintended pregnancy (EUP) exposes adolescent girls and young women (AGYW) to various social and financial hardships, negatively impacting access to resources and, thus, perpetuating gender-based inequities. School dropout is associated with poorer sexual and reproductive health (SRH) knowledge and, when coupled with suboptimal access to SRH services and persistent gender inequities, can elevate risks of EUP, HIV and other sexually transmitted infections, as well as sexual and gender-based violence (SGBV) [1, 2]. These factors trap women in a vicious cycle of poverty, inhibiting AGYW from harnessing their full potential. Investing in AGYW by building their agency and self-esteem and preventing educational attrition, are crucial cornerstones for achieving gender equity and fulfilling global and national human rights commitments, including the Sustainable Development Goals (SDGs).

Zambia has a youthful population, with adolescents under 18 years constituting over half (52.2%) of the population [3]. Adolescents are essential to Zambia’s socio-economic development but remain stifled by numerous health and development challenges. Nearly 10% of women report first marriage by age 15 (median age at first marriage: 19.1 years), and over a third are married by age 18 [4]. The age specific birth rate for 15–19-year-olds in Zambia hovers around 135 births per 1000 adolescents—an estimate towering over the global adolescent birth rate of 44 per 1000 [5].

The Ministry of General Education (MoGE) in Zambia noted a gradual increase in the number of teenage pregnancies among in-school girls. Between 2011 and 2019, a total of 120,878 pregnancies among in-school girls (109,850 in primary school and 26,752 in secondary school) were recorded [6–18]. Both in secondary and primary schools, pregnancies increased from 14,849 in 2012 [7], to 14,928 in 2013 [8], to 16,378 in 2014 [9]; in this three-year period, 46,155 pregnancies were recorded, a majority (83%) of which were among primary school-aged girls [8, 9]. In 2017 to 2018, a total of 28,669 girls got pregnant, the majority of whom (77%) where in primary school [10, 11, 17]. For many girls, adolescent childbearing signifies the end of education: only 49% of pregnant primary school girls returned to school in 2019 [7]. While there is nearly universal knowledge of contraceptive methods (99% of females have heard of any modern method, with no significant differences by age) [12], the high number of EUP requires the identification of strategies for translating the knowledge to preventive behaviuor. Among those aged 15–19 who have had a live birth and begun childbearing, the use of modern contraceptive methods is 41% [12].

To address the overlapping challenges of school attrition and EUP, innovative strategies for enhancing self-efficacy, agency, gender empowerment, and school retention are imperative. Comprehensive sexuality education (CSE), introduced throughout sub-Saharan Africa, including Zambia [13, 14], is a universally accepted, evidenced-based initiative for confronting the syndemics of EUP, HIV/STIs, and SGBV. The extant literature demonstrates that CSE reduces sexual risk behaviours and improves SRH knowledge [15], compared to abstinence-only education programs, which have shown little or inconclusive effects on these same indicators [16]. There is compelling evidence that in-school CSE leads to improved HIV/SRH knowledge, increased condom use, reductions in number of sexual partners, increased self-efficacy for HIV protection, more favorable attitudes towards safer sex, and delayed sexual debut [17]. A review of CSE evaluations found that programs addressing gender-based power dynamics, including raising awareness on human rights, were far more likely to achieve beneficial impacts on key health indicators (i.e., pregnancy, childbirth, HIV/STIs) than programs ignoring these topics, suggesting empowerment-based CSE approaches may be most impactful [18].

CSE implementation in Zambia, nevertheless, has confronted various operational and structural challenges. A small qualitative study in rural Nyimba District (Eastern Province) found that teachers implement the CSE curriculum with substantial discretion, which has resulted in censorship of specific elements of the curriculum deemed incompatible with local norms [19]. Another study in Kalomo District (Southern District) reported that despite parental support for CSE implementation, the lack of age-appropriate material in the CSE curriculum diluted SRH knowledge and information that pupils could glean from the curriculum [20]. In our own qualitative work in North-Western Province, we found that SRH information provided through school-based CSE insufficiently motivated SRH service-seeking for adolescents, who feared mistreatment by health providers and privacy violations [21]. Collectively, these barriers could synergistically attenuate the anticipated effectiveness of school-based CSE in Zambia. Efforts to strengthen CSE delivery in Zambia must, therefore, be responsive to identified implementation barriers, while taking into account, the findings from this paper.

Without access to quality health services, young people have limited ways to adopt or implement some of the practices or behaviours promoted by CSE curricula (e.g., condom use, strategies for pregnancy and HIV prevention). Efforts to make services more amiable and responsive to adolescents have largely failed to improve outcomes unless they are coupled with demand-generation activities [22]. There is less empirical evidence that looks simultaneously at instituting facility adjustments (i.e., health care provider capacity strengthening and sensitization) to cater to SRH needs of adolescents (on the supply side) and school-based activities to encourage their prompt healthcare-seeking (on the demand-generation side). To address this gap, this study developed and evaluated the effectiveness of a combination of in-school CSE and adolescent-responsive SRH services in Zambian primary and secondary schools, using a three-arm study design. In our approach, we recognized the sensitivity surrounding issues related to provision of comprehensive CSE in school, and thus complied with existing policies, while noting that each school acknowledged how they had integrated CSE in the school curriculum, based on provisions of the Ministry of Education; and we also convened initial meetings with the respective Parent Teacher Associations (PTAs).

The overall primary objective was to reduce EUP and improve SRH outcomes among AGYW in Zambia through increased access and utilization of SRH information and services.

## Methods

### Setting and study sample

The study was implemented in selected schools and health facilities in Solwezi and Mufumbwe Districts (North-Western Province) of Zambia from 2017 to 2020. These two districts were selected because of high background adolescent pregnancy rates [4]. A 2016 assessment among 15 schools in these two districts identified 1571 pregnancies in Solwezi District and 697 in Mufumbwe District among school-going AGYW [10].

A cross-sectional baseline assessment was conducted from August to November 2017 and followed by three subsequent rounds (R) of post-intervention (PI) data collection in November and December of each year: a first round (R1-PI) in 2018; a second round (R2-PI) in 2019; and a third and final round (R3-PI) in December 2020. At baseline, reported data on pregnancies at study sites were abstracted for 2016 and 2017.

The study sample was drawn from schools selected at baseline. Schools were purposively identified and matched for consistency, according to presence of teachers trained in CSE (through orientation seminars or workshops); student access to a government health facility offering SRH services, regardless of distance; comparable student populations by age: sex composition; and annual numbers of withdrawals due to pregnancy. Using these criteria, 12 schools were selected in Solwezi (six primary and six secondary) and 11 in Mufumbwe (five secondary and six primary). Each of the schools’ Parent Teacher Association gave permission for the study, prior to IRB approvals.

**Fig. 1:**
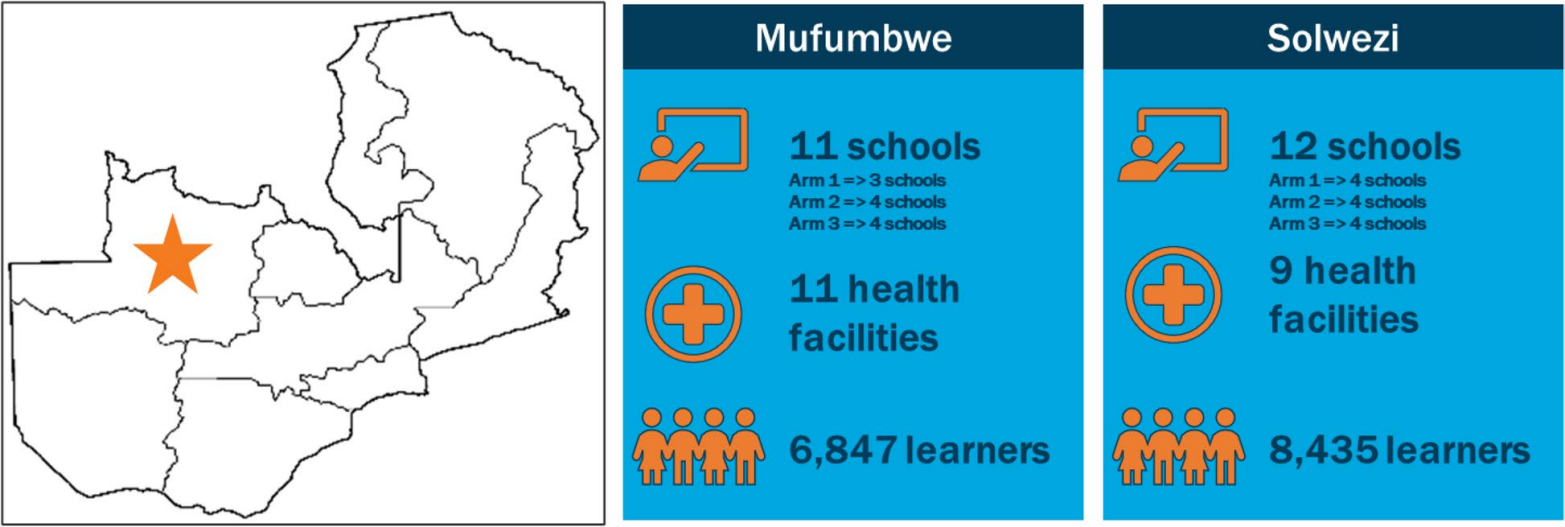
Distribution of schools in study districts

After baseline assessment, the 23 schools (see Fig. 1 above) were subsequently randomized into three study arms: Arms 2 and 3 served as the integrated services (“experimental”) groups, and Arm 1 served as the control (standard of care). Standard operating procedures (SOPs) to guide implementation were developed and shared with implementing partners, following detailed training on study interventions.

Prior to interventions, we engaged parents from all the study schools by convening meetings with the respective Parent Teacher Associations (PTA), and following our explanation of the study rationale, design and methods, all PTAs were unanimous in providing their approvals, in writing. Our study dissemination efforts have also included sharing of the data with the respective PTAs, community and political leaderships.

### Design and data collection

**Fig. 2:**
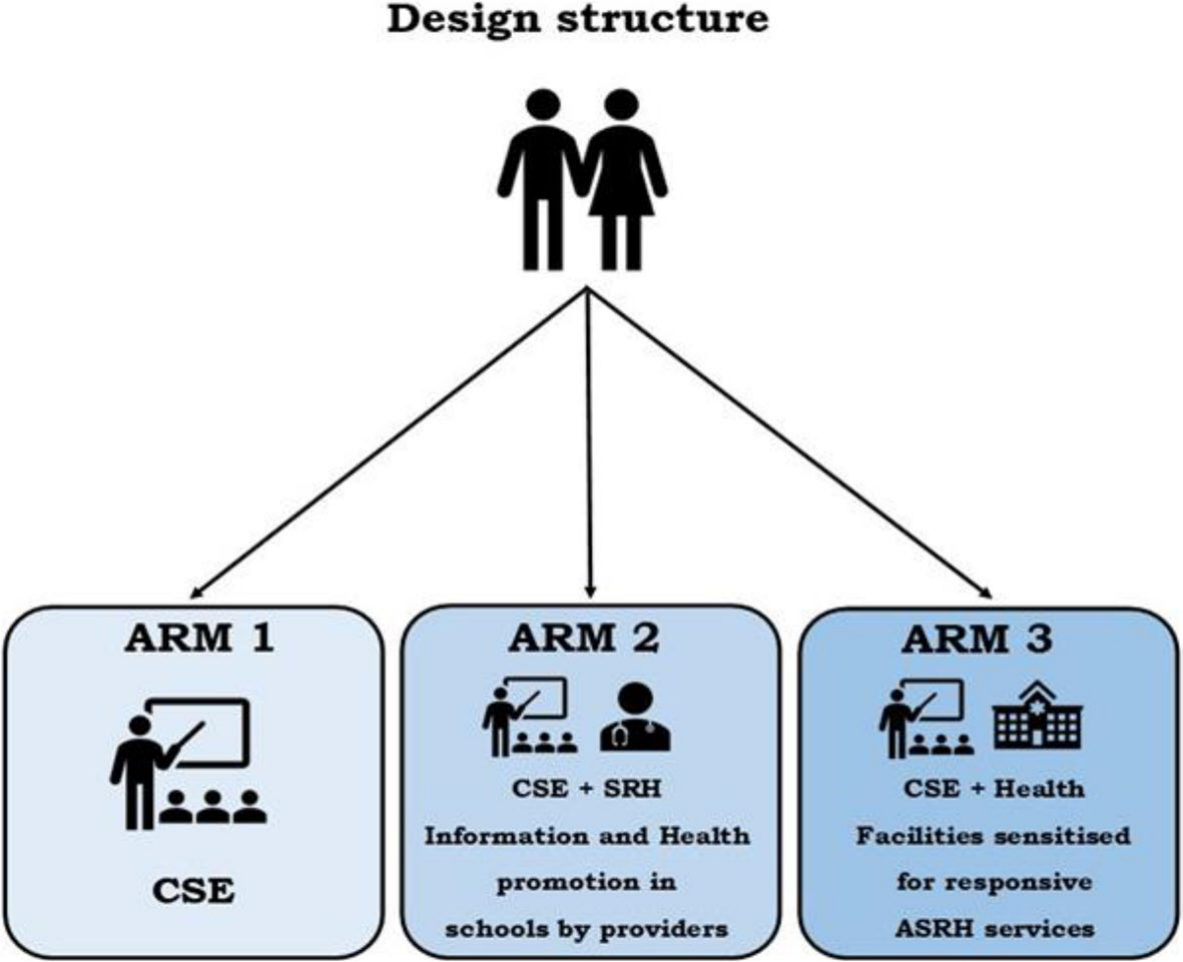
Summary schema of the study design

The three study arms each included the basic CSE curriculum, but with additional elements in Arms 2 and 3 (see Fig. 2 below):

**Arm 1 (CSE only control)** included routine provision of in-school CSE and availability of adolescent SRH services in facility catchment areas (per Ministry of Health (MoH) guidelines), with no additional interventions. Schools and health facilities in this arm continued CSE and adolescent SRH programs according to current government standards and regulations. The MoGE has implemented CSE teacher trainings (including in some teaching colleges) and has been training teachers on CSE and introduced CSE training in some teacher training colleges. Furthermore, the MoGE had printed the new curriculum books in which CSE had been integrated, and teachers were utilizing those books to teach CSE. In health facilities, the MoH had begun training health care workers on adolescent-friendly SRH services.

**Arm 2 (CSE + Health Outreach)** included school-based information on available adolescent health services, through health fairs by health providers and a referral slip system to facilities, to generate demand when need was expressed. In addition to CSE and SRH information in schools, catchment area health facilities were encouraged to be more accessible and responsive to adolescents, particularly those situated near schools. Cognizant of cultural and policy sensitivities related to SRH service provision, school-based SRH services information and referrals were offered at monthly school health fairs, as part of extramural activities, rather than during instructional periods. Thus, schools collaborated with local health facilities to host health fairs monthly on school premises. A school health committee, consisting of seven members including the Chair of the Parent Teacher Association, organized and coordinated health fairs. Services at health fairs were determined in collaboration with the MoH and MoGE and included: HIV counseling and testing, information on pregnancy prevention and pregnancy testing, contraceptive counseling, HIV prevention education, menstrual health and personal hygiene promotion, and hypertension and obesity monitoring.

For services unavailable at health fairs, including HIV or STI treatment, referrals were made to health facilities. To ensure adolescents completed any referrals made from the health fair to health facilities, the referral system included a de-identified referral slip that was completed by a health facility staff member, recorded at facility and returned to the school on a later date. Facility staff included in the referral process were either lay counselors or trained healthcare workers based at the facilities to which adolescents were referred.

**Arm 3 (CSE + Responsive Adolescent SRH Services)** included provision of health services outside of schools. In this arm, instead of providing information and services at schools, adolescent SRH services were provided at health facilities where staff were trained to be more responsive or receptive to adolescents’ SRH needs. In collaboration with the MoH, the project created a strong referral link between CSE-implementing schools and catchment area health facilities, to increase adolescents’ access to SRH services. In collaboration with the MoH, MoGE, and the Young Women Christian Association, monitoring visits were conducted at health facilities to evaluate compliance with SOPs. Schools were encouraged to forge partnerships with local healthcare facilities to create an enabling environment which discouraged stigmatization or discrimination of adolescents seeking health services they needed. Teachers were encouraged to support students who sought health services by directing them to facilities and encouraging them to adopt preventive health-seeking behaviors. Both teachers and healthcare workers were trained on available SRH services, being responsive and supportive to adolescents, so they could utilize services freely to fulfill their health needs. Services rendered at health facilities included: information on various SRH service topics like HIV/STI prevention, puberty, personal hygiene, menstrual health, and adolescent pregnancy prevention.

We also took into account potential barriers in accessing health services by in-school adolescent girls and boys, and convened the respective catchment areas’ health care providers, to sensitize them on the need to be more responsive and receptive to the SRH needs of adolescents and young people (AYP).

### Measures

**Fig. 3:**
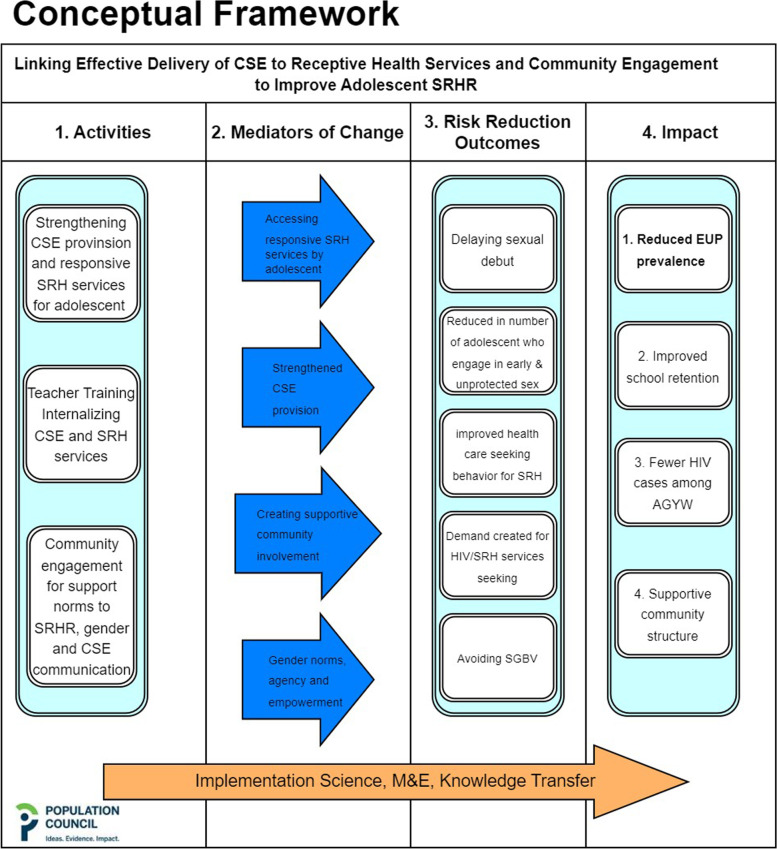
Protocol conceptual framework developed to guide the overall study conceptualization and implementation

The present paper is concerned with in-school EUP as the outcome of interest. In addition, background characteristics of the study population were collected annually at each questionnaire round. Data was collected using preloaded study instruments on tablets using SurveyCTO software. A conceptual framework (see Fig. 3) was developed to help explore potential pathways that reduction in EUP could be achieved. This paper explores the pathway that links provision of CSE and accessing responsive SRH services to reduction of EUP. Key elements in the conceptual frame entailed 1. Activities that ran concurrently, inclusive of teacher training on CSE that was being provided by the Ministry of Education throughout the country, but complemented with their orientation and updates on available health services on SRH that pupils could access, in intervention arm schools; pre-sensitized health services towards being more receptive to health needs of adolescents; and community engagement through the respective PTAs. 2. Hypothesizing, as mediators of change, an environment where health services in intervention school catchment areas are made more responsive to SRH information and service needs of adolescents that presented to them, aligned to strengthened provision of CSE, ensuring a supportive community environment through dialogue meetings with PTAs and including orientation discussions on gender sensitivities and empowerment to seek services, on introduction of interventions to pupils and teachers at each intervention school. 3. The conceptual framework made a number of risk reduction assumptions associated with intended outcome measures and, for this paper, analysis focused on reduced EUP, presumed on the combination of school and health facility interventions, within a supportive community environment. The study recognized that the timeframe to improve CSE provision and responsive health service are very different from the time frame to address and improve unequal gender norms, and thus avoided an attempt to factor this in the analysis of EUP, but attempts would be made, to ascertain gender related attitudes before and after the interventions. Thus, additional analyses evaluating the impact of differentiated CSE knowledge, behaviors and adolescent SRH service linkage models on other key outcomes, including gender norms, will be presented in subsequent manuscripts, alluding to this manuscript and our conceptual framework.

Data on annual pregnancies (number of pregnancies in enrolled primary or secondary school-going girls) from each school was collected at three time points in the post-intervention period, in the last quarter of each calendar year (2018, 2019 to 2020) and aggregated at the study arm level. Data on pre-intervention periods of 2016 and 2017 were also recorded. The study team, in collaboration with MoH and MoGE, abstracted data on pregnancies from each school’s guidance/counselling files and annual census reports during the monitoring visits.

#### Sample size

The overall sample size was calculated based on the number of adolescents required to derive differences, if any, in knowledge and behavioural variables that meet the criteria in each school and each selected grade, and thus more applicable in later analysis, while noting adequacy for lower-level variables, such as pregnancies. Thus, according to the Ministry of Education Statistical Bulletin of 2014, the estimated average number of students per classroom in North-Western Province is 55 for grades 1–7 and 45 for grades 8–12, with an average of two classroom streams per grade. Accounting for a 10% refusal rate, the minimum sample at each cross-sectional assessment was, therefore, 1704 adolescents, with upward adjustments to avoid in-classroom student discrimination, and maintaining enough power to test levels of significant differences in reported knowledge and sexual behaviors, irrespective of attrition due to in-school pregnancies.

$$\textrm{ss}=\frac{{\textrm{Z}}^2\ast \left(\textrm{p}\right)\ast \left(1-\textrm{p}\right)}{{\textrm{c}}^2}$$

Z = Z value (e.g., 1.96 for 95% confidence level)

p = percentage picking a choice, expressed as decimal (.5 used for sample size needed)

c = confidence interval, expressed as decimal.

#### Data management analysis

Collected data (entered using SurveyCTO) was transferred to Excel for preliminary cleaning, then transferred to Stata/IC 15.1 for further management and analysis. Bivariate logistic regression was implemented to examine whether socio-demographic characteristics in the study population differed significantly by study arm at endline (R3-PI). Within each study arm, paired t-tests were implemented to determine whether standardized pregnancy rates (number of pregnancies per 1000 AGYW enrolled at each school) changed significantly over the observation period. Guided by a difference-in-differences approach, we then implemented mixed-effects Poisson regression to determine whether observed changes in standardized pregnancy rates from baseline to endline differed significantly by arm, with the CSE-only arm serving as the referent group in analysis. Statistical significance was determined using a pre-specified alpha threshold of 0.95 (*p* < 0.05)*.*

## Results

### Background of sample socio-demographic characteristics

Descriptive sample characteristics at endline (R3-PI) of 986 participating AGYW are presented in Table 1. A total of 2114 respondents participated, 46.6%, (*n* = 986) of whom were AGYW and included in this analysis. Over half (54.8%) were aged 15–19 years (mean: 15 years, range: 12–24 years). Nearly all AGYW were Christian (92.8%). Most (65.8%) were in secondary school (grades 8 through 12), and one-third (34.2%) were in primary school (grades 5 through 7).

**Table 1:** Descriptive sample characteristics at endline (R3-PI)

	Arm			Total	*P* Value
	1	2	3		
	n (%)	n (%)	n (%)	n (%)	
**District**					
Solwezi	188 (54.5)	194 (54.8)	121 (42.2)	503 (51.0)	0.57
Mufumbwe	157(45.5)	160 (45.2)	166 (57.8)	483 (49.0)	0.08
**Age group**					
12–14 years	123 (35.7)	188 (53.1)	110 (38.3)	421 (42.7)	0.35
15–19 years	213 (61.7)	164 (46.3)	163 (56.8)	540 (54.8)	0.06
20–24 years	9 (2.6)	2 (0.6)	14 (4.9)	25 (2.5)	0.06
**Religion**					
Christianity	319 (94.9)	341 (96.9)	277 (96.5)	931 (96.1)	0.32
Others	17 (5.1)	11 (3.1)	10 (3.5)	38 (3.9)	0.60
**Education level**					
Primary school	90 (26.1)	159 (44.3)	88 (30.7)	337 (34.2)	0.08
Secondary school	225 (73.9)	59 (16.7)	24 (8.4)	123 (12.5)	0.06

Of those in secondary school, a greater proportion (21.1%) were in Grade 8 and Grade 9 (18.8%). There were no significant differences between the arms at endline, rendering them comparable.

### Pregnancies in school

**Fig. 4:**
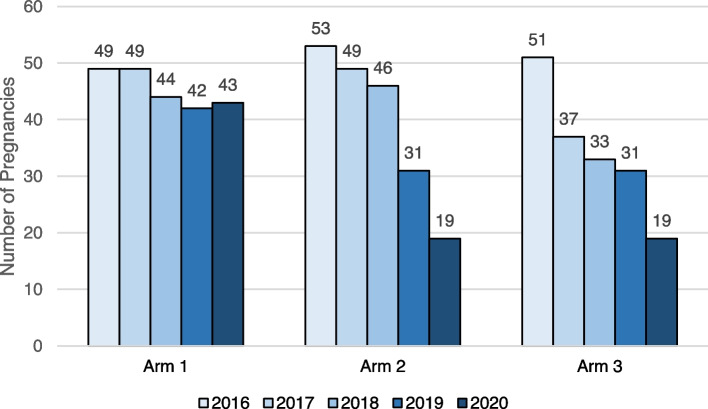
Cumulative number of school-level pregnancies, by year and study arm

By study endline in 2020 (R3-PI), there were noteworthy reductions in the numbers of adolescent pregnancies following exposure to the CSE linkage program, with marked reductions documented in Arms 2 and 3 (see Fig. 4 below), where periods 2016 was pre-intervention, and 2017 being formative and initiation of interventions.

A test of proportion was conducted to compare the pre-intervention (2016 and 2017) and post intervention (2020) trends in pregnancy (see Table 2). After comparing Arm1 (control) 2016 and 2017, there was no notable change recorded and the difference was not significant. However, Arms 2 and 3 recorded a significant change (*p* < 0.001 and 0.001, respectively), when compared at the pre- and post- intervention periods.

**Table 2:** Pregnancy test of proportion at pre and post interventions by arm

	Pre-Intervention (2016)	Post intervention (2020)	Difference	*P* Values
	n (%)	n (%)	N (%)	
Arm 1	1687 (3.0)	1, 588 (2.7)	99 (0.3)	0.606
Arm 2	2626 (2.0)	2, 561 (0.7)	65 (1.3)	0.001
Arm 3	1584 (3.0)	1, 422 (1.3)	162 (1.7)	0.001

At endline (P3-PI), Arm 1 recorded a pregnancy fraction of 2.70 per 100 AGYW (*n* = 43 pregnancies), whereas Arms 2 and 3 recorded pregnancy fractions of 0.74 per 100 AGYW and 1.34 per 100 AGYW, respectively. After conducting a test for proportion which compared schools in arms 2 and 3 to those in arm 1, results showed that schools in Arm 2 (*p* = 0.001) and Arm 3 (*p* = 0.001) reported significantly lower fractions of EUP relative to schools in Arm 1 (see Table 3).

**Table 3:** Proportion of enrolled schoolgirls who were pregnant in 2020

	# of Enrolled Schoolgirls	# of Enrolled Pregnant Girls	% Pregnant Girls, *P* value	
Arm 1	1, 588	43	2.70%	Reference
Arm 2	2, 561	19	0.74%	**0.001**
Arm 3	1, 422	19	1.34%	**0.001**

**Fig. 5:**
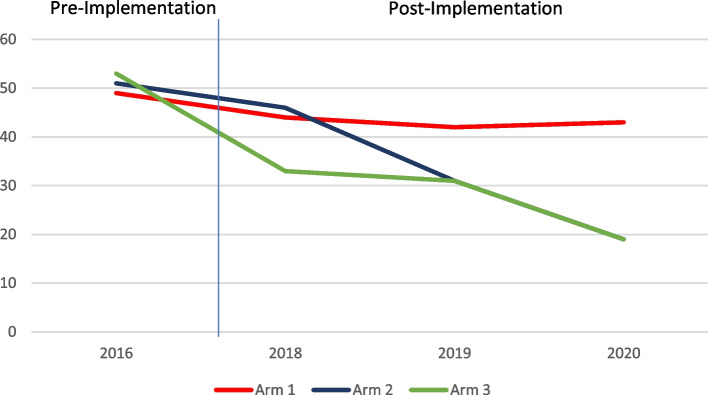
Schematic description of pregnancies over time in each arm, from which difference in difference tests were conducted

Figure 5 also shows the trends in pregnancy across arms over time. While schools in Arm 1 exhibited a steady rate in the number of annual pregnancies reported throughout the observation period, schools in Arms 2 and 3 reported steady reductions in the number of pregnancies among in-school AGYW following introduction of linked CSE with adolescent-friendly health services.

A difference-in-differences analysis was applied to compare the pre-intervention (2016) and post intervention (2020) IRR in pregnancy, between the control arm (Arm1 2016 and 2020) as compared with Arms 2 and 3, respectively, to estimate the relative measure of intervention effect (see Table 4). The reductions in pregnancy observed in Arm 2 (IRR 0.46, 95% CI 0.33–0.63, *p* < 0.001) and Arm 3 (IRR 0.66, 95% CI 0.51–0.84, *p* = 0.001), respectively, were significantly larger than the reductions in pregnancy observed in Arm 1.

**Table 4:** Differences in pregnancies between the arms before and after interventions

Study Group	Pre-Intervention (2016)			Post-Intervention (2020)			Pre-Post Difference				Difference-in-Differences	
	Pregnancies	Enrolled AGYW	Rate (per 1 k)	Pregnancies	Enrolled AGYW	Rate (per 1 k)	Pregnancies	Enrolled AGYW	Rate (per 1 k)	*p*	IRR (95% CI)	*p*
Arm 1	49	1687	29	43	1588	28	−6	−99	−1	0.949	1.00	*Ref*.
Arm 2	53	2626	20	19	2561	7	−34	−65	−13	0.174	**0.46 (0.33 to 0.63)**	**< 0.001**
Arm 3	51	1584	32	19	1422	13	−32	− 162	−19	0.142	**0.66 (0.51 to 0.84)**	**0.001**

Pregnancy metadata were also analyzed within each school to subsequently inform school-based targeted pregnancy reduction strategies, in conformity with implementation feedback SOPs. At R2-PI and R3-PI, schools in Arm 1 showed no notable reductions, with a high number of pregnancies in two schools, while in Arm 2, one school had a high pregnancy rate, and in Arm 3, there was concern at one school. Feedback was provided in each case, in line with developed implementation science research protocols.

## Discussion

Findings in this paper suggest a significant decline in early and unintended pregnancies within schools featuring integrated CSE linked to SRH information and services, particularly when efforts are made to bring information on SRH services, to schools and linking CSE with health facilities that are sensitized to be more receptive to the ASRH needs of AGYW who may or not be in school. Although there are disparities in achievements, evidence from this paper suggests that pregnancies declined in the two intervention arms, but with room for additional improvement. Although in-school pregnancies were documented across arms following trial implementation, there were significantly greater declines in EUP in the two intervention arms relative to the control arm, which were associated with CSE linked to SRH services and information.

Adolescent pregnancy remains a major public health concern, even when there is one pregnancy, it disrupts the learning opportunity of the adolescent, with potential negative health and social consequences. CSE, integrated in the school curriculum, confers opportunities for imparting scientifically accurate information about SRH. Despite having moderate SRH knowledge and acceptability of SRH services, few sexually experienced AGYW utilize SRH services, resulting in high EUP. Multiple reviews and studies across a diverse range of settings in Europe, the United States, Africa, and Mexico confirm that CSE contributes to preventing unintended adolescent pregnancies by providing students with knowledge, life skills, and information on contraceptive options. It can reduce early sexual debut and prevent early and unintended pregnancy among AGYW [23]. However, few studies have examined the impact of linking school-based CSE implementation with peripheral SRH services on in-school pregnancies [24–26]. Our study is, therefore, among the first to measure the impact of integrating adolescent sexual and reproductive health services into school-based CSE.

A noteworthy finding of this study was that in-school pregnancies did not decline significantly in the CSE-only control arm, which underscores the role of combining CSE implementation with information on available and responsive SRH services. Several studies in Zambia have reported barriers to CSE implementation, including parental objections, perceived incompatibility of curricular material with social norms, inadequate training and support for teachers, instructor discomfort with material or self-efficacy delivering lesson plans, and inattention to health systems-related factors impacting SRH service provision to adolescents and SRH outcomes [19–21]. In the context of prolonged CSE implementation, the absence of a significant effect on pregnancy reductions could indicate that school-based CSE alone, as implemented, may be insufficient to addressing adolescents’ SRH needs. However, when coupled with SRH services information and service referral at school, and provision (facility-based), pregnancy reductions were notably significant. The effectiveness of CSE implementation may, therefore, hinge upon successful integration with SRH service provision that is adolescent-friendly and attentive to adolescent needs.

According to the Ministry of General Education in Zambia [7], between 2011 and 2018, up to 65,726 in-school girls got pregnant and dropped out of school, with the majority (80.4%) having been still in primary school [6–11], and contributing to a high national figure of 28.5% among those aged 15-19 yrs. in 2018 [4]. Pregnancy is an indicator that adolescents are having unprotected sex, which puts them at risk of HIV and other STIs. Pregnancy also leads to other vulnerabilities among adolescent girls, including child marriage, possibility of resorting to unsafe abortions, and subsequent maternal health and child births complications. Despite such high rates of in-school pregnancies reported in sub-Saharan Africa [27], evidence-based programmes to curb the high rates have been limited, and no systematic programmes that proactively promote linkages between schools offering CSE and health facilities, to assist adolescents to access and utilize SRH services, have been reported. This study was therefore the first to develop and test models that support such linkages.

Teenaged mothers are at higher risk of adverse pregnancy outcomes or complications, and children born to very young mothers are at increased risk of sickness and early death [28]. Women who are mothers at an early age have lower rates of participation in the labour force, difficulty finding employment, and incurring lower earnings. Their children typically have reduced earning potential as well, which perpetuates the cycle of poverty from one generation to another. Therefore, there is need for continued messaging on the multi-purpose use of condoms for prevention of EUP, HIV/AIDS, and STIs, when sexually active, to break the poverty cycle. Studies show that CSE has immense potential to provide young people with the necessary information about their bodies and sexuality, to reduce misinformation, shame and anxiety, and to improve their abilities to make safe and informed choices about their sexual and reproductive health and ultimately reduce EUP [29–31].

Sometimes shortages of essential SRH commodities and supplies are a disincentive in accessing and using SRH services in the public sector. Our mitigation approach to this has been through engaging the political leadership, including parliamentary committees, to share our findings, with a view to show and highlight the cost benefits of investing in SRH commodities, whose down-stream consequences, if neglected, far outweigh the initial costs of availing them. Such consequences include high school dropouts, unsafe abortion, maternal mortality and morbidity, needless contribution to high total fertility, and poverty through curtailed economic growth targets.

While there could be gaps in the provision of CSE in some schools, based on individual ideological convictions, we did not encounter individual resistances from schools, possibly attributed to the support we received from the combination of Ministry of Education, Ministry of Health and the respective PTAs. During our pre-study orientations, we implored teachers at each school to shun from personal beliefs in approaching the issues of CSE and accessing SRH services by their pupils, in times of need.

The study has a number of policy implications for consideration, with a view to reduce in-school pregnancies and allow AGYW to realize their full potential. These include the following:

- The need for policy decision makers and programme directorates in Ministries of Education, Health and Economic Planning and Finance to work together to facilitate access to CSE that is linked to SRH services and information;
- The need for constant dialogues and engagement of PTAs by Ministries of Education and Health, on the issues of preventing EUP in schools and encouraging links between provision of CSE and accessing health care.
- The need for constant orientation of health services and sensitization of health care providers by Ministries of Health, to be more responsive and receptive to the needs of AYP, including in-school pupils.
- The need for relevant civil society organizations or Development partners, or Ministries of Health, to engage in interactive health education outreach programmes in schools, which place emphasis on prevention of EUP, among other health and social wellbeing issues, such as menstrual hygiene and respectful gender relations.

Our study is not without limitations. First, while our primary outcome of interest was early and unintended pregnancy, we also collected data on background characteristics of respondent pupils at each school, other key process or outcome measures, like SRH and gender equity knowledge and behaviours, which could be among mediating factors in the relationship between intervention implementation and pregnancy outcomes. However, to avoid overwhelming this paper, whose explicit measure is pregnancy outcome by study arm intervention, these multiple factors and related outcomes will be the focus of subsequent analyses of these data. Second, pregnancy data were derived from school administrative records, which may not have accurately captured all pregnancies, particularly if AGYW dropped out of school and did not report pregnancy to the school, as the reason for dropping out. However, this limitation would apply to all study arms. Lastly, the unique implementation climate of CSE in North-Western Province and health systems context in Zambia may attenuate the generalizability of our findings outside Zambia. Future research should examine the impact of comparable CSE-linked interventions in other contexts, especially in other countries in the region, affected by high school attrition from EUP.

## Conclusion

Considering that the odds of exposure to both in-school CSE and accessible receptive health facility-based SRH services, significantly increased for students in intervention arms 2 and 3, and pregnancies significantly declined, it appears that at least both of those additional interventions are beneficial and should be implemented in these and additional schools. However, to compensate individual intervention study limitations and enhance outcomes, a hybrid intervention arm incorporating both intervention elements would be ideal.

In addition, further research, which employs qualitative study designs, will be required to establish the circumstances surrounding the individual in-school pregnancies, as well as health and social outcomes affecting the girls, especially among those who fail to return to school. A potential study limitation was having to rely on school records on pregnancies that occur in a calendar year, as that misses early unitended pregnancies occurring at the end of the year, but the ideal of including pregnancy tests, is not feasible, and this limitation is countered by the potential for such late pregnancies to be randomly distributed across the three study arms.

## Data Availability

The datasets generated and /or analysed specific to the current reported study are available on reasonable request to Population Council, Zambia, Data Manager Mr. Kondwani Kasonda kkasonda@popcouncil.org.

## Abbreviations

AGYW: Adolescent girls and young women
AYP: Adolescents and young people
CSE: Comprehensive sexuality education
EUP: Early and unintended pregnancies
HIV: Human immunodeficiency virus
IRR: Incident rate ratios
SDGs: Sustainable development goals
SGBV: Sexual gender-based violence
SRH: Sexual and reproductive health
STI: Sexually transmitted infections
